# P08 Optimizing ward-based antimicrobial stewardship by empowering pharmacy technicians to complete IV to oral reviews

**DOI:** 10.1093/jacamr/dlae217.012

**Published:** 2025-01-23

**Authors:** Shazia Nazir, Amy Long, Fiona Robb

**Affiliations:** Leeds Teaching Hospitals NHS Trust, Leeds, UK; Leeds Teaching Hospitals NHS Trust, Leeds, UK; Leeds Teaching Hospitals NHS Trust, Leeds, UK

## Abstract

**Background:**

At Leeds Teaching Hospitals (LTHT), antimicrobial stewardship (AMS) is a critical yet challenging initiative. With a diverse range of specialties and departments across multiple sites, we face barriers such as high patient volumes, limited reviews and frequent staff turnover. Effective AMS is essential to ensure antibiotics are used appropriately, improving patient outcomes, reducing healthcare costs and minimizing healthcare-associated infections (HCAIs). Prolonged IV antibiotic use can delay discharge, increase costs, heighten infection risks, demand more nursing time, and raise the likelihood of HCAIs. Therefore, embedding strong AMS principles throughout the trust is key to optimizing care. In a previous project, I successfully demonstrated the benefits of 48–72 h reviews for patients on IV antibiotics. Building on this success, I proposed delivering targeted teaching sessions to pharmacy technicians across the trust, spanning various specialties. As most wards at LTHT have pharmacy technician coverage, this presented an opportunity to enhance AMS efforts across the trust. By equipping pharmacy technicians with the knowledge and skills to support IV-to-oral switches (IVOS), we could significantly amplify the impact of stewardship strategies.

**Methods:**

Pharmacy technicians from various specialties were trained to conduct virtual reviews using an electronic IV antimicrobial review tool. Patients were identified using an Emeds report, which provided data on daily antimicrobial prescribing. The list was filtered to include patients who had been on IV antibiotics for 48–72 h. *Inclusion criteria:* Patients aged 16 years and older, inpatients prescribed IV antibiotics for 48–72 h. *Exclusion criteria:* Patients younger than 16 years, those on antibiotics for cystic fibrosis, on directed antimicrobial therapy, on oral antibiotics, or receiving prophylactic antimicrobials. Data collection took place from December 2022 to March 2023 as part of a baseline audit. The audit covered adult wards across both hospital sites, and results were analysed using Excel.

**Results:**

The audit included 87 patients. IVOS recommendations were made for 38 of these patients, and 30 of the recommendations were followed. As a result, 17 patients were discharged within 48 h of the switch. For patients whose clinical condition had not improved, the recommendation was to continue IV therapy, which clinicians adhered to. Overall, 83% of patients had documented care plans aligned with pharmacy technician advice. This intervention demonstrated a positive impact on AMS by reducing unnecessary IV antibiotic use and increasing the documentation of clinical reviews and actions.

**Conclusions:**

This audit highlights the potential of involving pharmacy technicians in AMS initiatives to improve prescribing practices. Increased participation of pharmacy technicians in daily patient reviews resulted in more IVOS therapy transitions and enhancing patient outcomes. Collaboration with infection prevention teams and pharmacy technicians will be crucial to further optimize AMS within the trust. By making AMS a shared responsibility, we can collectively improve patient care and reduce antibiotic resistance across LTHT.

